# Omics approaches to understanding the efficacy and safety of disease-modifying treatments in multiple sclerosis

**DOI:** 10.3389/fgene.2023.1076421

**Published:** 2023-01-30

**Authors:** Lorena Lorefice, Maristella Pitzalis, Federica Murgia, Giuseppe Fenu, Luigi Atzori, Eleonora Cocco

**Affiliations:** ^1^ Multiple Sclerosis Center, Binaghi Hospital, ASL Cagliari, Department of Medical Sciences and Public Health, University of Cagliari, Cagliari, Italy; ^2^ Institute for Genetic and Biomedical Research, National Research Council, Cagliari, Italy; ^3^ Dpt of Biomedical Sciences, University of Cagliari, Cagliari, Italy; ^4^ Department of Neurosciences, ARNAS Brotzu, Cagliari, Italy

**Keywords:** multiple sclerosis, genomics, transcriptomics, proteomics, metabolomics, disease-modifying treatments, efficacy, safety

## Abstract

From the perspective of precision medicine, the challenge for the future is to improve the accuracy of diagnosis, prognosis, and prediction of therapeutic responses through the identification of biomarkers. In this framework, the *omics* sciences (genomics, transcriptomics, proteomics, and metabolomics) and their combined use represent innovative approaches for the exploration of the complexity and heterogeneity of multiple sclerosis (MS). This review examines the evidence currently available on the application of omics sciences to MS, analyses the methods, their limitations, the samples used, and their characteristics, with a particular focus on biomarkers associated with the disease state, exposure to disease-modifying treatments (DMTs), and drug efficacies and safety profiles.

## Introduction

Multiple sclerosis (MS) is a chronic autoimmune disease of the central nervous system (CNS), characterized by pathological, clinical, and neuroradiological complexity, that has variable prognoses and disease outcomes (Filippi et al., 2016). As epidemiologic studies have previously shown, MS rates vary with several genetic and environmental factors (Ward and Goldman, 2022); many of these driving the phenotypic (Olsson et al., 2017) and neuroradiological heterogeneity (Lorefice et al., 2019a) of MS and the different trajectories of MS progression. Changes in diagnostic methods and criteria, incorporating imaging and spinal fluid abnormalities in patients presenting with a clinically isolated syndrome, have allowed earlier diagnoses of MS (Thompson et al., 2018) and, improved disease control and management. MS occurs more frequently in young adulthood and is characterized by an initial relapsing–remitting phase (RRMS) followed by evolution to secondary progressive MS; about 10% of patients present a progression from the onset of the disease, which usually begins later in these cases (Filippi et al., 2016). At the extremes, the paediatric forms (before age 18), in which the clinical onset is closer to the biological onset (Margoni et al., 2022), and the late onset MS (after age 50), in which the opposite happens (Naseri et al., 2021), fuel the debate on the role of the environment, lifestyle, and other factors in the clinical onset of the disease, the biological reserve and brain resilience, and the long-term disease progression. There is a strong debate on how MS patients acquire disabilities, either through relapse-associated worsening (more attributable to the inflammatory aspects of the disease), or through progression independent of relapse activity (Lublin et al., 2022). On clinical grounds, it is of central importance to distinguish between patients with more inflammatory characteristics and those with more neurodegenerative aspects, for the early identification of MS progression (Filippi et al., 2020) and to better guide disease management and therapeutic choices; in the current scenario, specific options are available for progressive forms (Amezcua, 2022). In the last decade, the therapeutic armamentarium of MS has changed to include the introduction of disease-modifying therapies (DMTs) with different mechanisms of action, routes of administration, levels of efficacy, and safety profiles (Faissner and Gold, 2022). In this context, the identification of biomarkers that could shed light on the mechanisms of inflammation and neurodegeneration, and reveal the biological mechanisms involved in the response and safety of DMTs, represent a crucial challenge in the therapeutic decision-making process. This makes medicine more personalised. Within this framework, omics technologies are emerging as innovative approaches for studying the complexity of MS (Villoslada and Baranzini, 2012), including the numerous factors influencing its evolution and response to immunotherapies. Omics sciences can improve the matching of a patient with the best available DMT among the varied therapeutic scenarios and drugs, which have different effects and mechanisms of action, and help determine the best “window of therapeutic opportunity” (Cocco et al., 2015).

Given this scenario, this review summarizes how emerging multi-omic approaches (genomics, transcriptomics, proteomics, metabolomics and others) can improve the understanding of MS, also discussing the available evidence on biomarkers, which may predict the efficacy and safety of DMTs, and the effects of MS-unrelated factors on MS evolution.

## Omics approaches

Systems biology approaches have shown promise in elucidating the mechanisms underlying complex diseases through the integration of different levels of omics information, using a holistic perspective that considers all processes involved and their dynamics (Villoslada and Baranzini, 2012). Ideally, this would facilitate the discovery of different types of biomarkers and the identification of how they are related across the different levels of biological complexity (genes, molecules, cells, tissues, organisms) to the clinical phenotype (Baranzini, 2006). There are various branches of omics sciences, from the more-known genomics, transcriptomics, proteomics, and metabolomics, to several subcategories of disciplines in biology, all with names ending with the suffix *-omics*. Omics approaches and their combinations have grown enormously, offering new evidence on the inflammatory and neurodegenerative mechanisms of MS, as well as on possible predictors of its evolution. The main omics sciences, the current methods on which they are based, and the evidence available to date are described below, with particular detail on understanding the MS pathogenetic processes, efficacies of DMTs, and safety implications.

### Genomics

Genomics was the first omics approach used on a large scale and has benefited from advances in microarray technology, next-generation sequencing (NGS), and genome-wide association studies (GWAS). Through an agnostic study of the entire genome, genomics has expanded the knowledge of the genetic bases of complex diseases and quantitative traits, including immune traits and drug responses. These data create new opportunities for drug discovery and the selection of molecular biomarkers predictive of drug response and efficacy. The genetics of MS have been extensively investigated over the past 15 years through several large GWAS that have advanced the understanding of genetic predisposition to MS. Currently, over 233 unequivocal, independent, genetic associations have been identified (International Multiple Sclerosis Genetics Consortium: Hafler et al., 2007; Sawcer et al., 2011; Beecham et al., 2013; De Jager et al., 2009; Patsopoulos et al., 2011; Andlauer et al., 2016; Sanna et al., 2010). They are located throughout the genome and include 32 independent variants in the HLA region and one on the X chromosome. The greatest MS association from the GWAS data is for the HLA-DRB1*15:01 risk allele (International Multiple Sclerosis Genetics Consortium, 2019a). Most of the genes involved in MS susceptibilities are expressed mainly in immune cells (B and T lymphocytes, natural killer [NK] cells, monocytes, macrophages) and microglia (International Multiple Sclerosis Genetics Consortium, 2019b); risk variants are often located on promoters and enhancers. Disease risk variants can be combined to quantify genetic predisposition, stratify individuals according to disease risk, or predict prognostic outcomes and response to therapy (Lambert et al., 2019). Mathematical models combine information from a large number of associated genetic variants by using a weighted sum of allele counts, in which the weights reflect the estimated effect size (odds ratio) of disease-associated alleles to generate polygenic risk scores (PRS). PRS have been used as predictive biomarkers in high-risk individuals for several diseases, and their inclusion in clinical care has been proposed (Khera et al., 2018). However, there is currently no MS-predictive score model with potential clinical utility. Several authors have tested predictive models that include both genetic risk and known environmental risk and other variables, such as gender and age (De Jager et al., 2009; Shams et al., 2022). However, the inherent complexity of the disease, characterised by a large number of variants that contribute to the disease with small or moderate effects (Odd ratio three 1.1 and 1.5) and with a large share of genetic heritability that still remains unexplained, is probably the main reason for the difficulty in developing a predictive score can provide accurate predictions at the individual level.

A complete list of associated variants and summary statistics for each study is available (https://www.ebi.ac.uk/gwas/).

One of the open challenges is to systematically combine genomic data with other omics data. For this purpose, co-localization methods are increasingly being used (Giambartolomei et al., 2014). The coincidence of associated variants that influence disease risk and modulate quantitative variables or regulate gene expression, “coincident associations”, provide information on disease mechanisms and inform drug targeting of specific pathways (Floris et al., 2018).

It is known that the use of genomics in drug development improves their success and reduces development time (Plenge et al., 2013). This is supported by retrospective analyses (Cook et al., 2014; Nelson et al., 2015) showing that drugs developed to target molecules identified by genetic associations are more likely to pass efficacy testing.

In addition, genomics research retrospectively linked a variant on TNFSF13B with an augmented risk of MS and systemic lupus erythematosus (LES) and a blood increase in soluble BAFF, which is already a target of belimumab, a monoclonal antibody approved for LES (Stohl and Hilbert, 2012) and in clinical trials for MS (Steri et al., 2017).

The increasing availability of genetic data collected by international biobanks (Wu et al., 2019) and linked to information on drug-action phenotypes promote GWAS on genetic effects related to drug responses (pharmacogenetics). These can provide useful information on the efficacy and safety of treatments that are directly applicable to clinics.

For example, individuals can be stratified into responders and non-responders, or avoid side effects, based on known genetic factors (personalized medicine). In the United States, Europe, and Asia, many initiatives are studying personalized medicine models based on preventative screening of a large number of pharmacogens (PGx-testing). Preliminary economic evaluations of efficacy have demonstrated a broad benefit from genotype-guided treatments.

While many studies have been published, indicating a large number of drugs-variant associations, most analyse small cohorts and show small effect sizes; these are unlikely to be replicated in other studies. Unfortunately, studies on MS drugs remain limited, and genomics studies have not yet led to significant changes in clinical practice toward personalising therapy. Interferon-beta (IFN-β) is a first-line therapy in MS, that is, used widely (Tsareva et al., 2016). Unfortunately, up to 50% of patients show a suboptimal response to IFN-β and are categorized as non-responders. IFN-β is the most studied biomarker associated with the treatment response. Many GWAS in different populations are conducted; however, these are limited to relatively small cohorts of patients (Mahurkar et al., 2017). Anti-IFN-β antibodies may develop during treatment. These antibodies may reduce both the bioactivity and clinical efficacy of IFN-β, reducing or abrogating treatment effects (Pachner et al., 2009). For this reason, many genomics studies have looked for genetic variants that influence the production of anti-IFN-β antibodies, identifying some associated HLA SNP variants, as reported in Table 1 (Andlauer et al., 2020). In addition, glatiramer acetate (GA), another first-line treatment of MS, shows variability in response among patients (Carter and Keating, 2010), and GWASs have found a multi-SNP (rs80191572, rs28724893, rs1789084 and rs139890339) signature specific to GA identifies MS patients with a greater response to treatment in multiple and independent cohorts (Ross et al., 2017). Pharmacogenetic studies on the response of other treatments in MS patients are lacking. In contrast, many studies on rituximab in rheumatological and haematological diseases suggest that the outcome of anti-CD20 therapy could be predicted by SNPs that affect the cytotoxic function of macrophages and NK cells and B-cell survival (Zhong et al., 2020).

**Table 1:** Genetic variant and allele risk for response to interferon beta and anti-drug antibody measurement traits in multiple sclerosis from the Andlauer TFM Study (Andlauer TFM et al., 2020)

Variant and risk allele	P-value	P-value annotation	RAF	OR	Beta	CI	Mapped gene
rs9281971-TTTTTTT	2 × 10-17		0.36	-	0.6 unit decrease	[-0.46-0.74]	HLA-DQA1
rs9272775-C	2 × 10-19		NR	-	0.40715143 unit increase	[0.32-0.5]	HLA-DQA1
rs17205731-T	2 × 10-11	(conditional on HLA-DRB1*04:01)	0.05	-	0.62026006 unit increase	[0.44-0.8]	HLA-DQB1, MTCO3P1
rs3129783-G	4 × 10-19		0.44	-	0.3 unit decrease	[-0.24-0.36]	HLA-DQB1, MTCO3P1
rs886401-A	4 × 10-12		NR	-	0.24661802 unit increase	[0.18-0.32]	CCHCR1
rs9271721-G	5 × 10-22		NR	-	0.32949224 unit increase	[0.26-0.4]	HLA-DQA1, HLA-DRB1
rs9281962-T	5 × 10-32		0.44	-	0.5091026 unit increase	[0.42-0.59]	HLA-DQA1, HLA-DRB1
rs1265086-T	5 × 10-9	(conditional on rs9272071 + rs28746882)	0.38	-	0.15234222 unit decrease	[-0.1-0.2]	POLR2LP1, CCHCR1
rs2523608-G	2 × 10-13		0.48	-	0.18175249 unit increase	[0.13-0.23]	HLA-B
rs9260765-C	5 × 10-11		0.45	-	0.17372645 unit increase	[0.12-0.23]	MICD, HLA-W
rs9272071-C	5 × 10-18		0.32	-	0.22615278 unit decrease	[-0.17-0.28]	HLA-DQA1
rs28746882-A	5 × 10-11	(conditional on rs9272071)	0.08	-	0.27678168 unit increase	[0.19-0.36]	HLA-DQB1, MTCO3P1
rs9272775-C	3 × 10-24		0.23	-	0.41 unit increase	[0.33-0.49]	HLA-DQA1
rs559242105-TA	5 × 10-10	(conditional on rs9272775)	0.18	-	0.28 unit decrease	[-0.18--0.38]	HLA-DPA2, COL11A2P1
rs9268633-A	1 × 10-17		0.39	-	0.2 unit increase	[-0.0392-0.0392]	HLA-DRA, TSBP1-AS1
rs9271673-C	2 × 10-28		NR	-	0.37 unit increase	[0.31-0.43]	HLA-DQA1, HLA-DRB1
rs9281971-TTTTTTT	2 × 10-17		NR	-	0.16 unit decrease	[-0.12-0.2]	HLA-DQA1
rs559242105-TA	1 × 10-8	(conditional on rs28366299)	0.18	-	0.85 unit decrease	[-0.56-1.14]	HLA-DPA2, COL11A2P1
rs28366299-A	7 × 10-19		0.19	-	1.27 unit increase	[1.0-1.54]	HLA-DQA1, HLA-DRB1
rs77278603-A	2 × 10-24		NR	-	1.06 unit increase	[0.86-1.26]	HLA-DRB5, HLA-DRB9
rs9271700-G	5 × 10-27		0.42	-	1.25 unit increase	[1.01-1.49]	HLA-DQA1, HLA-DRB1
rs522308-T	2 × 10-15		0.25	2.6	-	[2.05-3.29]	HLA-DQA1, HLA-DRB1
rs2454138-A	5 × 10-19		0.22	-	0.41 unit increase	[0.31-0.51]	HLA-DQA1, HLA-DRB1
rs2454138-A	8 × 10-19		0.22	-	0.41 unit increase	[0.31-0.51]	HLA-DQA1, HLA-DRB1
rs2454138-A	1 × 10-18		0.22	-	0.41 unit increase	-	HLA-DQA1, HLA-DRB1

Drug treatments can have substantially different adverse reactions in individuals, due to genetic variants that modulate individual responses to the drug. The US Food and Drug Administration site (https://www.fda.gov/drugs/science-and-research-drugs/table-pharmacogenomic-biomarkers-drug-labeling) and PharmGKB (http://www.pharmgkb.org) maintain an up-to-date list of genetic variants implicated in drug efficacy and safety. In a search for DMTs used in MS treatment, only clinically actionable variants (level of evidence 1A or 1B) were found between CYP2C9 and siponimod. Other variants with type 3 levels of evidence (annotation based on a single significant result) were found for IFN-β, GA, and rituximab (Table 2).

**Table 2:** Genetic variants implicated in efficacy and safety of MS drugs

LEVEL	VARIANT	GENE	**DRUGS**	**PHENOTYPE CATEGORIES**
Level 1A	CYP2C9*1, CYP2C9*2, CYP2C9*3	CYP2C9	siponimod	Metabolism/PK
Level 3	rs12044852	CD58	interferon beta-1a, interferon beta-1b	Efficacy
Level 3	rs760316	FHIT	interferon beta-1a, interferon beta-1b	Efficacy
Level 3	rs10760397	GAPVD1	interferon beta-1a, interferon beta-1b	Efficacy
Level 3	rs2291858	GAPVD1	interferon beta-1a, interferon beta-1b	Efficacy
Level 3	rs10819043	GAPVD1	interferon beta-1a, interferon beta-1b	Efficacy
Level 3	HLA-B*15:01:01:01	HLA-B	interferon beta-1a	Efficacy
Level 3	rs9272105	HLA-DQA1	interferon beta-1a, interferon beta-1b	Efficacy
Level 3	HLA-DRB1*04:01:01	HLA-DRB1	interferon beta-1a	Efficacy
Level 3	rs2205986	IRF6	interferon beta-1a, interferon beta-1b	Toxicity
Level 3	rs4774388	RORA	interferon beta-1a	Efficacy
Level 3	rs10494227	ZNF697	interferon beta-1a, interferon beta-1b	Efficacy
Level 3	rs4278350		interferon beta-1a, interferon beta-1b	Efficacy
Level 3	rs1448673		interferon beta-1a, interferon beta-1b	Efficacy
Level 3	rs3133084		interferon beta-1a, interferon beta-1b	Efficacy
Level 3	rs75041078		glatiramer acetate	Toxicity
Level 3	rs12459996		glatiramer acetate	Toxicity
Level 3	rs1056854		glatiramer acetate	Toxicity
Level 3	rs2229109	ABCB1	cyclophosphamide, prednisone, rituximab,	Toxicity

Major initiatives are needed to study the genetic variability in the MS drug response, with the aim of identifying rare variants that affect safety. To expand the list of biomarkers that can be used in precision medicine, studies that integrate omics sequencing data with other epigenetic and metabolomics data are also necessary.

New long-read sequencing technologies may have great potential to enrich and improve the knowledge of genetic factors that play a role in variable drug responses (van der Lee et al., 2022) Long-read sequencing also offers a comprehensive characterization of variants, including structural and rare variants. In addition, haplotype problems can be overcome, contributing to improved combinations of variants implicated in drug efficacy and safety.

### Epigenomics

Epigenomics is a depth approach that studies epigenetic mechanisms at whole genome level - i.e. all those chemical changes in the DNA that do not make any changes to the nucleotide sequence - at whole genome level. Epigenetic changes cooperate with genetic mechanisms to determine transcriptional activity and, while somatically heritable, are also reversible and can arise as a consequence of environmental factors. DNA methylation, histone modification and microRNA, associated post-transcriptional gene silencing, are three key epigenetic mechanisms.

Epigenetic contribution to MS susceptibility was already considered more than a decade ago to explain the low concordance (25%–30%) of MS in monozygotic twins (Ebers et al., 1986), who possess a genetic similarity of 100%. Also, the higher MS prevalence in women compared to men and the transmission disequilibrium of the HLADRB1*15 risk allele from mother to daughter (Chao et al., 2010), indicates an effect probably mediated by epigenetic mechanisms.

For this reason, numerous epigenetic studies have been conducted, however, many of these have included a limited number of patients, sometimes obtaining conflicting results. In this review, we describe some representative whole-genome epigenetic studies (EWAS), as reported in Table 3.

**Table 3:** Main results for epigenomics and trascriptomics

EPIGENOMICS					
Sample	Year	Study	Method	Characterization	Gene or main result reported
CD4+ T cells	2014	Graves, M et al,.	methylation arrays	Status of disease	HLA-DRB1
CD4+ T cells , CD8+ T cells	2015	Maltby, V. E. et al.	methylation arrays	Status of disease	different epigenetic profiling of CD8+ T cells and CD4+ T cells
CD8+ T cells	2015	Bos, S. D. et al.	methylation arrays	Status of disease	hypermethylation in MS respect to controls
CD4+ T cells	2017	Maltby, V. E. et al.	methylation arrays	Status of disease	HLA-DRB1 (hypomethylation), HLA-DRB5 (hypermethylation)
PBMC	2016	Kulakova, O. G. et al.	methylation arrays	Disease progression	different methylation in PPMS than in RRMS
monocytes	2018	Kular, L. et al.	methylation arrays	Status of disease	HLA-DRB1 (DRB1*15:01 )
PBMCs (monozygotic twins )	2020	Souren NY, et al.	methylation arrays/bisulfite sequencing	Status of disease and Drug response	TMEM232, ZBTB16 RSAD2, MX1, IFI44L and PLSC
Blood	2017	Pinto-Medel, M.J., et al.	methylation arrays	Clinical activity, Drug response	LINE-1
CD4+ T-cells	2021	Roostaei T. . et al.	methylation arrays	Status of disease	colocalized MS-cis-mQTL effects: rs59655222 rs12478539, rs438613, rs7731626, rs67111717, rs4896153, rs55858457, rs354033, rs7855251, rs4939490, rs12365699, rs405343, rs34947566, rs3809627, rs1077667, rs1465697, rs6032662, rs2248137, rs760517. colocalized MS-trans-mQTL effects rs3809627
CD4+ T-cells	2018	Maltby, V. E. et al.	methylation arrays	Status of disease	methylated genes associated at MS risk:
					SLC44A2, LTBR, CARD11 e CXCR5
CD4+ T cells	2016	Sanders et al.	RNA-seq	Disease progression	miR-21-5p, -26b-5p, -29b-3p, -142-3p and -155-5p)
Blood	2018	Liguori M. et al.	RNA-seq	pediatric MS	let-7a-5p, let-7b-5p, miR-25-3p, miR-125a-5p,.miR-942-5p, miR-221-3p, miR-652-3p, miR-182-5p, miR-185-5p, miR-181a-5p, miR-320a, miR-99b-5p, miR-148b-3p

TRANSCRIPTOMICS					
Sample	Year	Study	Method	Characterization	Gene or main result reported
Blood	2020	Ye F et al.	Microarray	Disease progression	(FTH1, GBP2, MYL6, NCOA4, SRP9
	2018	Gafson AR et al	RNA-seq	Drug response	Nrf2 pathway, NFkB pathway
	2016	Cordiglieri C et al	Gene expression	Drug response	ITGA2B, ITGB3, CD177, IGJ, IL5RA, MMP8, P2RY12, S100β
	2014	De Felice et al.	RNA-seq	Drug response	mir-26a-5p
	2014	Moreno-Torres I et al.	RNA-seq	Drug response	FOXP3, GPI, and FCRL1 and NK bright and plasmablasts
	2014	Parnell GP et al.	RNA-seq	Drug response	S6 protein
	2014	Hecker M et al.	RNA-seq	Drug response	miR-29 family

Graves, M et al., indicated DNA methylation association at HLA-DRB1 locus in CD4^+^ T cells related to MS risk (Graves et al., 2014). Another study found different epigenetic profiling of CD8^+^ T cells and CD4^+^ T cells in MS patients (Maltby et al., 2015; Kiselev et al., 2022), and the same authors indicated hypomethylation in CD4^+^ T cells at HLA-DRB1 and HLA-DRB5 hypermethylation in a MS cohort (Maltby et al., 2017). Maltby VE and others, in 2018 by means of an epigenome-wide association analysis of DNA methylation in CD19^+^ B-lymphocytes from 24 relapsing-remitting MS patients undergoing various treatments and 24 healthy controls, observed a large differentially methylated region in the lymphotoxin alpha (LTA) locus and four other MS-associated genes: SLC44A2, LTBR, CARD11 and CXCR5, suggesting that B-cell-specific DNA-methylation may be associated with MS risk. (Maltby et al., 2018). Roostaei et al. studing the DNA methylation profiles of primary CD4^+^ T-cells from MS patients, disclosed a broad map of cis-mQTL (methyl quantitative trait), and identified 19 M susceptibility loci with colocalised cis-mQTL effects, such as the TBX6 locus, which also has an effect in trans. (Roostaei et al., 2021).

Bos S. and others found evidence for DNA hypermethylation in CD8^+^ T cells of MS patients respect to controls (Bos et al., 2015). Kulakova, O. G. et al. found differential DNA methylation in peripheral blood mononuclear cells (PBMC) on relapsing-remitting MS (RRMS) and primary-progressive MS (PPMS) patients respect healthy controls, reporting more methylation changes on PPMS than in RRMS (Kulakova et al., 2016). Another study, which analyzed DNA methylation in monocytes from MS patients, confirmed the methylation at the HLA-DRB1 locus and demonstrated that homozygous DRB1*15:01 patients showed significantly lower levels of methylation at the HLA-DRB1 locus than heterozygous patients and non-carriers (Kular et al., 2018). A recent study, examining methylation in PBMCs of 45 monozygotic twins discordant for MS, have identified two differentially expressed regions associated with MS - TMEM232 promoter and ZBTB16 enhancer. Additionally, differentially methylated regions located in the RSAD2, MX1, IFI44L and PLSCR1 genes, which are upregulated in the blood cells of IFN-treated MS patients, thus indicating biomarkers for monitoring the effects of IFN treatment in PBMCs (Souren et al., 2019). Pinto-Medel, M.J., and others found that individuals with high methylation levels of LINE-1 have an increased risk of MS. Furthermore, treated MS patients with high levels of LINE-1 methylation showed an increased risk of clinical activity. The authors also propose global DNA methylation levels as a possible biomarker for differential clinical response to IFNβ (Pinto-Medel et al., 2017).

Also, many miRNA studies have also been conducted on MS patients using both peripheral blood and cerebrospinal fluid, but again these studies often have small sample sizes and lack reproducibility. However, there are a small number of studies using high-throughput methods investigating the expression of circulating miRNAs in the context of the difference in miRNA profile between RRMS and SPMS subtypes and in drug response.

Sanders et al. (2016) using next-generation sequencing (NGS) to profile miRNA expression in CD4^+^ T cells of SPMS patients, identified 42 dysregulated miRNAs. Five of these (miR-21-5p, −26b-5p, −29b-3p, -142-3p and -155-5p) showed downregulated expression and had the potential to be used as diagnostic biomarkers of SPMS (Sanders et al., 2016). Another study, which used NGS sequencing approach in pediatric MS patients, identified several significantly upregulated miRNAs (let-7a-5p, let-7b-5p, miR-25-3p, miR-125a-5p, miR-942-5p, miR-221-3p, miR-652-3p, miR-182-5p, miR-185-5p, miR-181a-5p, miR-320a, miR-99b-5p) and one downregulated miRNA (miR-148b-3p) in pediatric MS patients compared to pediatric controls. The targets of this dysregulates miRNA are genes linked to immunological functions (TNFSF13B, TLR2, BACH2, KLF4), as well as genes involved in processes related to autophagy (ATG16L1, SORT1, LAMP2) and ATPase activity (ABCA1, GPX3) (Liguori et al., 2018).

Overall, these studies indicate that several distinct epigenetic signatures have been detected in different populations of peripheral immune cells, supporting the hypothesis of the involvement of epigenetic factors in the development of MS.

Epigenomics can help to unravel complex gene regulatory interactions, better understand pathogenetic mechanisms and optimize MS treatment. However, to our knowledge no epigenetic biomarker is currently in a clinical.

### Transcriptomics

Transcriptomics is an approach that allows for the comprehensive and extensive study of RNA transcripts in a group of cells or a specific cell. It is based on the use of high-throughput methods such as microarray analysis and NGS RNA-sequencing (RNA-seq). Comparison of transcriptomes in selected subgroups (cases and controls, different cell populations, different treatments) enables the identification of differences in gene expression, so-called *gene expression signatures*, which can be used for prognostic and disease-predictive purposes or to predict responses to drug treatments.

Gene expression signatures have been especially useful in cancer and have entered clinical practice; e.g., to establish prognoses and personalize therapy in early breast cancer (Puppe et al., 2020). Several studies investigating gene expression profiles in the peripheral blood of MS patients have been published. These identified peripheral gene signatures associated with both disease and its progression (Ye et al., 2020) and drug response (Hecker et al., 2013; De Felice et al., 2014; Moreno-Torres et al., 2014; Parnell et al., 2014; Cordiglieri et al., 2016; Gafson et al., 2018), as summarized in Table 3, suggesting that gene signatures have the potential to identify individuals at risk of relapse. Ye F et al. showed a five-gene signature (FTH1, GBP2, MYL6, NCOA4, SRP9) used to calculate risk scores to predict individual predicting relapse-free survival (Ye et al., 2020). Cordiglieri C et al. described a gene signature, that include ITGA2B, ITGB3, CD177, IGJ, IL5RA, MMP8, P2RY12, S100β genes, associated with positive response in RR-MS and drug immunomodulatory effects (Cordiglieri et al., 2016). The Gafson AR et al. study showed expression changes for genes involved in Nrf2 pathway activation and NFkB pathway inhibition, which are associated with the clinical and mid-term response to dimethylfumarate (DMF) (Gafson et al., 2018). Another study, which used RNA-seq technology, indicated a different gene expression signature (FOXP3, GPI, and FCRL1), and distribution of subpopulations of lymphocytes (NK bright and plasmablasts) in MS patients who were responsive to fingolimod compared to non-responders. The authors proposed a predictive model include that a combination of cellular, molecular and clinical markers (EDSS and gender) are possible response biomarkers (Moreno-Torres et al., 2018). RNA-seq technology has also been used to analyse the whole blood transcriptome of untreated and IFNβ-treated MS patients (Parnell et al., 2014), indicating a downregulation of S6 protein in IFNβ responders.

De Felice B et al., in their research, showed a significant change in the expression level of mir-26a-5p at different stages of treatment in RR responder MS patients treated with INF-β. They hypothesised that mir-26a-5p might downregulate the expression level of genes related to glutamate signalling in MS patients treated with INF-β and point to it as a possible biological marker to predict the identification of INF-β responders during therapy, thus reducing ineffective treatments (De Felice et al., 2014). Another study profiled microRNA (miRNA) expression changes in response to IFNβ and found a specific downregulation of the miR-29 family (Hecker et al., 2013). Other miRNA results are reported in the epigenetics sections.

However, many publications are only descriptive and need further studies with larger numbers of patients to validate the proposed models. To our knowledge there are no transcriptomic biomarkers validated for clinical use in MS. RNA-seq studies also provide information on alternative splicing and give a quantitative assessment of genotype influence on gene expression (quantitative expression loci: eQTL). Integration of eQTL, GWAS, and phenotype association data (PheWAS) is useful for detecting the effects of genetic variants on cis- and trans-expression levels of genes (Orru et al., 2020) and to indicate causative genes whose products can be used as therapeutic targets. In MS, genes that code for a therapeutic target have been detected through the integration of genomic data, phenotypes, and eQTLs; these include BAFF (Amezcua, 2022) or CD40, MERTK, and PARP1 (Jacobs et al., 2020). The same approach can inform the repurposing of existing drugs for new therapeutic indications. Large expression datasets in different human tissues are currently made available by the Genotype-Tissue Expression Project (GTEx); recent publications show that expression data from specific tissues integrated with other omics data can inform the prediction of drug side effects (Duffy et al., 2020). A more-detailed transcriptome characterization, down to the single cell, is now possible thanks to single-cell technologies (scRNA-seq). This monocellular technology allows the study of methylation, chromatin, and proteomics at the same level of resolution. The integration of these omics data will not only make new drugs available but also improve prognostic models on disease and drug efficacy and safety.

## Proteomics

Proteomics is the branch of biomedical studies that specifically analyses an organism’s entire protein content, including functions and interactions. *Clinical proteomics* analyses the role of proteins as disease biomarkers; *functional proteomics* evaluates the role of proteins in pathological and physiological processes; both are growing fields of interest in the exploration of complex diseases such as MS (Drabik et al., 2007). Several technologies are used in proteomic approaches, including chromatography-based techniques, enzyme-linked immunosorbent assay (ELISA), mass spectrometry, and gel electrophoresis (Drabik et al., 2007). Several biological samples can be examined for quantitative proteomic analysis in neurological conditions, including blood, saliva, tears, urine, and other biological fluids (Sandi et al., 2022); testing limits are linked to the isolated nature of the CNS, which thereby limits the exploration of MS. Cerebrospinal fluid (CSF) is of extreme interest, although it is not readily available. Several attributes of proteomics make it an attractive approach for exploring MS; for example, evaluating the role of proteins/peptides as effector molecules in physiological and pathological processes. Several proteomics studies have been conducted utilizing animal models of MS, and some studies were performed on human tissue samples (Sandi et al., 2022; Li et al., 2018). A recent CSF proteomic analysis of MS patients and neurological controls identified several hundred proteins (approximately 300) that changed significantly; pathway analysis associated these with various biological processes including inflammation, cell adhesion, and the immune response (Kroksveen et al., 2017). Recently, a study based on serum/plasma samples established that subjects with pre-symptomatic MS differed from the control group in the expression of 22 proteins involved in the complement and coagulation pathways, as well as in lipid transport (Wallin et al., 2015). Our previous study using quantitative analysis of salivary peptides in a mass spectrometry-based top-down proteomic approach found different levels of 23 proteins (subtypes of cystatin, statherin, antileukoproteinase, and prolactin-inducible protein) that may distinguish between MS and control groups; the results are consistent with the inflammatory condition and altered immune response typical of the pathology (Manconi et al., 2018). In addition, we observed reduced oxidation of S-type cystatins, which represented the larger portion of cystatins found in our saliva samples, highlighting the role of brain oxidative stress and the oxidant/antioxidant balance in inflammation and neurodegeneration in MS (Manconi et al., 2018). Regarding the discovery of potential markers of interest for disease activity, studies have been performed on the blood and CSF of patients with RRMS forms. Complement C4b increases in the CSF of active MS (Li et al., 2011). In addition, an increased level of the complement C4a fragment during relapses was found in the sera of relapsing patients, with a decrease found in phases of remission (Sawai et al., 2010). Another study identified the up- or downregulation of several proteins related to blood vessel development (protein 14-three to three, metavinculin, myosin-9, plasminogen, reticulocalbin-2 and-3, ribonuclease/angiogenin inhibitor 1, annexin A1, tropomyosin, and Ras-related protein Rap-1A) as potential new markers of active MS disease. This indicates they have a role in dysregulation of the blood–brain barrier and, thus, on the migration of activated leukocytes responsible for the development of demyelinating lesions of MS (Alexander et al., 2007). Interestingly, Tremlett et al. previously explored the protein signatures of MS phenotypic groups at the extremes of progression (benign and aggressive cases of MS), identifying panels of serum biomarkers of MS progression related to inflammation, opsonisation, and complement activation (Tremlett et al., 2015). However, research using proteomic analysis to discover biomarkers for MS progression is rare, and these data require further confirmation. Similarly, few studies have explored the effect of different DMTs on the proteome of patients with MS intending to identify potential biomarkers that can predict treatment response. Previously, De Masi et al. assessed the potential of proteomics for discriminating between IFN-treated patients and untreated RRMS patients; a down-expression of cortactin and fibrinogen β chain precursor in the blood samples of the treated MS group was reported, suggesting a pharmacological response to IFN (De Masi et al., 2013). In patients exposed to natalizumab, nine proteins showed decreasing levels in plasma; phosphatidylethanolamine-binding protein 1 (PEBP1) and reticulon 3 (RTN3) levels had the most significant changes, particularly in a group of patients with less disability progression (Bedri et al., 2019). These findings are very preliminary and reveal little about the treatment efficacy or the progressive aspects of the disease.

### Metabolomics

Metabolomic research has emerged as a promising approach for identifying potential biomarkers in complex and heterogeneous diseases such as MS. Through the detailed analysis of the metabolites detected in a biological sample, it may be possible to identify a fingerprint of MS. If the dynamic multi-parametric responses ongoing in an individual at any given time exhibit metabolites attributable to inflammatory or other pathways, these may characterize MS status (Cocco et al., 2016). Three analytical techniques are commonly used in metabolomics: gas chromatography coupled to mass spectrometry (GC–MS), liquid chromatography coupled with single-stage mass spectrometry (LC–MS), and nuclear magnetic resonance (NMR) spectroscopy; the latter can simultaneously identify multiple metabolites, is exceptionally reproducible, and has a fast measurement time requiring minimal sample preparation. Moreover, by combining different analytical techniques with targeted and untargeted approaches, more expansive and comprehensive metabolomic investigations of MS can be conducted (Jafari et al., 2021). Various studies have evaluated biofluids as novel metabolomic biomarkers, to improve diagnoses, patient stratification, and therapy choices. The most-investigated biofluids were CSF, blood, and urine; the latter two in particular because of their easy availability. Conversely, CSF testing is more invasive, but it is considered the “gold standard” fluid in MS because it reflects the inflammatory processes of the CNS. These biofluids contain different metabolites and, for reasons including their composition and availability, they have different potentials for providing insight into the diagnosis, disease activity, and progression of MS.

Previously, Reinke et al. used NMR spectroscopy analysis to investigate a set of CSF metabolites between MS patients and healthy controls. They found a reduction in 3-hydroxybutyrate, citrate, phenylalanine, 2-hydroxyisovalerate, and mannose in MS-derived CSF samples; these metabolites suggest alterations to energy and phospholipid metabolism (Reinke et al., 2014).

More recently, significant differences in amino and fatty acids in the CSF of patients with newly diagnosed MS were identified using LC-MS; in relationship with the inflammatory disease activity, the most significant changes were observed in levels of arginine, histidine, and palmitic acid. These findings highlight the importance in MS pathogenesis of fatty acids, which form part of myelin, and of some amino acids, and show a close connection to immunological processes of the disease (Židó et al., 2022). Consistent with these findings, the analysis of the CSF and the serum of patients with RRMS and the primary progressive course, which was previously assessed by our group, allowed the identification of several altered metabolites (lipids, biogenic amines, and amino acids) involved in various metabolic activities of interest, including energy metabolism and tryptophan biosynthesis (Murgia et al., 2020; Herman et al., 2019). Moreover, pathway analysis indicated the metabolism of glutathione, nitrogen, glutamine–glutamate, arginine–ornithine, phenylalanine biosynthesis, tyrosine, and tryptophan as the main discriminants between the two phenotypic classes (Murgia et al., 2020). Interestingly, some studies have focused on the evaluation of metabolomic biomarkers associated with disease phase and activity (Simone et al., 1996; Lutz et al., 2007; Kim et al., 2017). Lutz reported a significant relationship between the CSF lactate concentration and the number of inflammatory MS brain plaques; the β-hydroxyisobutyrate level was related to the presence of these plaques (Lutz et al., 2007). Similarly, high CSF lactate levels were found in MS patients during clinical exacerbation and on magnetic resonance imaging (MRI) of Gd-enhanced plaques by Simone, suggesting that changes in lactate levels may depend on anaerobic glycolytic metabolism in activated leukocytes during the inflammatory phases of MS (Simone et al., 1996). Analogously, metabolic changes principally related to altered energy metabolism and fatty acid biosynthesis, with isoleucine and valine being downregulated in MS relapse compared to MS remission, were described by Kim et al. on ^1^H-NMR spectra of CSF samples (Kim et al., 2017). Several studies also evaluated the blood of MS patients to assess its metabolomic signature, its clinical phenotype, and the disease activity, both using NMR (Mehrpour et al., 2013; Cocco et al., 2016) and mass spectrometry (Lazzarino et al., 2017, Lim et al., 2017, Bhargava et al., 2018; Nourbakhsh et al., 2018; Villoslada et al., 2017; Kasakin et al., 2019; Stoessel et al., 2018; Fitzgerald et al., 2021; 80; Levi et al., 2021; Singhal et al., 2018). Using NMR analysis of plasma samples of 73 patients with MS (therapy-free for at least 90 days) and 88 healthy controls, we reported lower levels of glucose, 5-OH-tryptophan, and tryptophan, while the levels of 3-OH-butyrate, acetoacetate, acetone, alanine, and choline were increased in the MS group (Cocco et al., 2016). These findings highlight the importance of energy metabolism and phospholipid metabolism for MS processes, consistent with the results of a study that identified the involvement of glucose metabolism in MS (Mehrpour et al., 2013). Among the studies with the largest sample sizes carried out using mass spectrometry, Villoslada et al. demonstrated a robustness of sphingomyelin and lysophosphatidylethanolamine for discriminating between healthy controls and patients with MS; the levels of several other metabolites (hydrocortisone, glutamic acid, tryptophan, eicosapentaenoic acid, ^13^S-hydroxyoctadecadienoic acid, and lysophosphatidylcholines) were more associated with disease severity (Villoslada et al., 2017). Overall, these results point to an imbalance in MS of the phospholipid and sphingolipid composition of the serum, as well as changes in several amino acids such as glutamic acid or tryptophan; these metabolites could be involved in the activation of the immune system or reflect changes in the CNS composition due to myelin destruction (Villoslada et al., 2017). A more extensive study conducted by Fitzgerald identified striking abnormalities in aromatic amino acid metabolites, with a broad shift of these toward oxidative pathway metabolites that are also reported to be strongly associated with MS disability (Fitzgerald et al., 2021). Also consistent with prior studies, changes in tryptophan pathway metabolites, with lower circulating levels of both tryptophan and its endogenous metabolites (e.g., kynurenine) were described in MS as compared to healthy controls (Fitzgerald et al., 2021). Consistent with these findings, an NMR urinary metabolic signature for MS has been recently described, with alterations of metabolites involved in energy, fatty acid metabolism, and mitochondrial activity being able to distinguish MS from neuromyelitis optica–spectrum disorder (Gebregiworgis et al., 2016). As can be seen from the results reported above, findings vary according to the samples examined (e.g., CSF, plasma, serum, urine), the patients included in the study (RRMS or progressive MS), and the associations that are being evaluated in regards to disease activity, level of disability, *etc.* The question becomes even more complicated when we investigate the effects of immunotherapies on the metabolomic profile of MS patients and the associations with disease activity and treatment response. To date, few studies have evaluated this aspect. By performing mass spectrometry analysis on plasma samples, Bhargava et al. showed that DMF treatment alters lipid metabolism and that changes in fatty acid levels are related to DMF-induced immunological changes (Bhargava et al., 2017). In particular, metabolic changes induced by DMF treatment are related to changes in the absolute counts of lymphocyte and CD8^+^ T cell subsets; however, these preliminary data cannot yet predict the effectiveness of DMF therapy (Bhargava et al., 2018). Later, it was shown that GA influences the metabolic profile of MS subjects, inducing a reduction of lactate and tyrosine and an increase of some oxidative phosphorylation markers: citrulline, ornithine, and tryptophan. This returns the MS metabolic profile to that of healthy subjects, especially in patients that show a full response to treatment (Signoriello et al., 2020). Similarly, as reported in our previous study, IFN-β therapy acts on the MS metabolomic profile, resulting in profiles similar to those of healthy controls, with acetoacetate, acetone, 3-hydroxybutyrate, glutamate, and methylmalonate levels significantly decreasing during treatment, whereas tryptophan levels increase (Lorefice et al., 2019b). In addition, differences in the baseline metabolome between responder and non-responder patients were found in the levels of lactate, acetone, 3-OH-butyrate, tryptophan, citrate, lysine, and glucose, indicating that it is potentially possible to identify the baseline metabolomic profile that predicts a better response to treatment (Lorefice et al., 2019a). Furthermore, metabolomics has also been used for predicting the development of neutralizing anti-drug antibodies in MS patients treated with IFN-β, which contributes to predicting the immunogenicity against IFN-β (Waddington et al., 2020) associated with the loss of treatment efficacy. Our group previously investigated the ability of metabolomics to predict DMTs safety, by using the metabolomic approach to evaluate whether patients who started treatment with fingolimod had a basal metabolic profile predictive of the subsequent response to treatment and cardiac complication at the first dose. Differences were observed in metabolites predominantly involved in the synthesis and degradation of ketone bodies, glycolysis and gluconeogenesis, and propanoate metabolism in patients who presented with a cardiac complication at the first dose; this highlights the potential of this approach for selecting the best candidates for this therapy (Lorefice et al., 2017). Table 4 details the main metabolomic studies performed on MS patients, also reporting the few studies aimed at evaluating the effects of DMTs on metabolomic signatures. The main challenge of the future will be to integrate a metabolomics approach with other omics sciences to predict the efficacy and safety of various DMTs in the context of the modern vision of precision medicine.

**Table 4:** Summary of metabolomics studies performed on MS patients

METABOLOMIC CHARACTERIZATION OF MS SUBJECTS				
Sample	Year	Study	Platform	Metabolomic characterization
CSF	1996	Simone et al.	NMR	MS and Non-MS
	2007	Lutz et al.	NMR	CIS and Non-MS
	2014	Reinke et al.	NMR	MS and Non-MS
	2017	Kim et al.	NMR	MS , HC, NMOSD
	2019	Herman et al.	Mass Spectrometry	MS and HC; MS Phenotypes
	2020	Murgia F et al.	NMR	RRMS and PPMS
	2022	Židó M, et al	HPLC	MS and HC
SERUM	2013	Mehrpour et al.	NMR	MS and HC
	2017	Lim et al.	Mass Spectrometry	MS and HC; MS Phenotypes
	2017	Lazzarino et al.	HPLC	MS and HC; MS Phenotypes
	2017	Villoslada et al.	Mass Spectrometry	MS and HC
	2018	Nourbakhsh et al.	Mass Spectrometry	Pediatric MS and HC
	2021	Fitzgerald et al.	Mass Spectrometry	MS and HC; MS Phenotypes
	2021	Levi et al.	Mass Spectrometry	MS and HC
PLASMA	2018	Singhal et al.	Mass Spectrometry	MS and HC
	2018	Bhargava et al.	Mass Spectrometry	MS and HC
	2018	Stoessel et al	Mass Spectrometry	MS and HC; MS Phenotypes
	2019	Kasakin et al.	Mass Spectrometry	MS and HC
URINE	2016	Gebregiworgis et al.	NMR	MS, HC, NMOSD
METABOLOMIC FEATURES RELATED TO DMTs				
SERUM	2018	Bhargava et al.	Mass Spectrometry	Dymatilfumarate Exposure
	2019	Lorefice et al.	NMR	Interferon Beta Exposure
	2020	Waddington et al.	NMR	Interferon Beta Immunogenicity
	2020	Signoriello et al.	NMR	Glatiramr Acetate Exposure
	2017	Lorefice et al.	NMR	Fingolimod Safery

### Lipidomics

**FIGURE 1:**
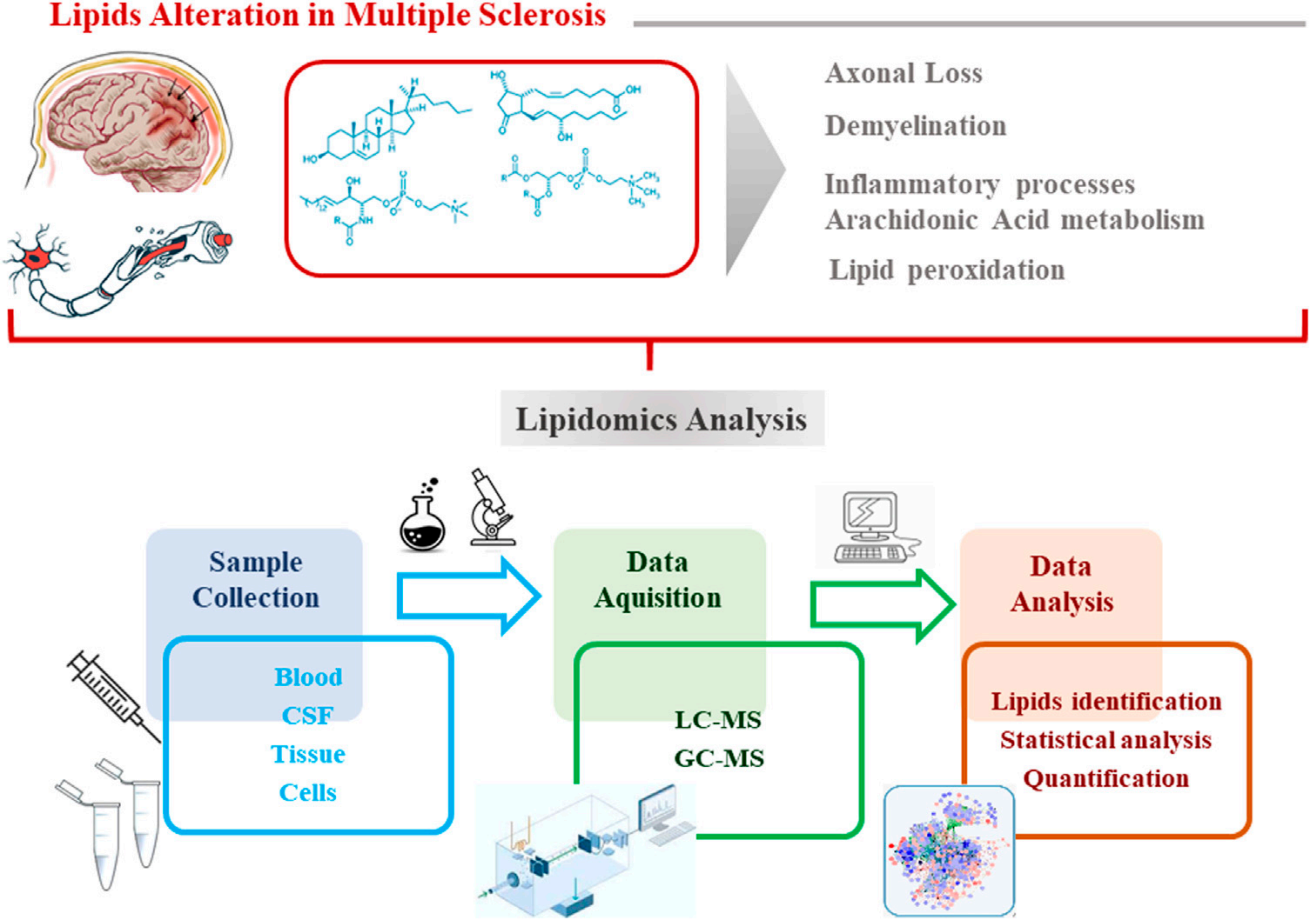
Workflow of a typical lipidomics experiment:sample preparation, instrumental analysis, data processing, to data interpretation.

Lipids represent an important class of biomolecules involved in different vital cellular processes (Züllig et al., 2020). In addition, several pieces of evidence suggest a significant role played by lipids in the MS background, as alterations in their metabolism contribute to MS pathogenesis and to disease severity (Ferreira et al., 2020). Considering the complexity and heterogeneity of the lipid classes (lipidome), high-throughput lipidomics analysis is a suitable approach to evaluate the variation of lipids at a molecular level (Ferreira et al., 2020). The method of choice for analyzing lipid molecules or massive assemblies is certainly mass spectrometry (both GC and LC), due to its sensitivity and specificity (Rustam and Reid, 2018). The workflow of a typical lipidomics experiment starting to the sample preparation, instrumental analysis, data processing, to data interpretation is represented in Figure 1. The most common biological matrices used for the investigation of the lipidome in MS are serum, plasma and CSF. Before the instrumental analysis, sample are treated according to specific protocols which provide: i) the reduction of the complexity of the sample; ii) discarding unwanted non-lipid compounds; iii) the enrichment of the analytes of interest (lipids).

Lipids can be considered a hallmark of demyelination and repair as they are substantially involved in the myelin sheath formation. For example, myelin sheath of oligodendrocytes contains polyunsaturated fatty acids (PUFA) and is highly vulnerable to lipid peroxidation, a dangerous process in MS pathogenesis as it can stimulate apoptotic events (Ibitoye et al., 2016). Studies have demonstrated that PUFA react with reactive oxygen species (ROS, products by processes of oxidative stress, common feature in MS) generating a cascade of oxidative damage leading to cell death (Jana and Pahan, 2007). Several studies found increased levels of lipid peroxidation products and ROS in CSF and plasma of MS patients (Haider et al., 2011; Ibitoye et al., 2016), together with mitochondrial damage, this latter often mediated by ceramides (Cer), another class of lipids (Halmer R et al., 2014).

Lipids are also involved in cell signaling, in communication, and in transport in the CNS (Pousinis et al., 2020) as well as inflammatory processes (Reale and Sanxhez-Ramon, 2017). In this regard, the metabolic pathway of arachidonic acid (AA) is overactivated in the CNS of MS patients (Palumbo, 2017). AA is a component of the bilayer of cellular membranes. Elevated concentrations of ROS and cytokines in MS (Rajda et al., 2017) give rise to its release leading to the production of pro-inflammatory compounds, such as prostaglandins and leukotrienes, that are upregulated in this pathology and seem to be involved in the pathogenic mechanisms of demyelination, axonal pathology and oligodendrocyte loss, contributing to the development of motor disabilities.

Changes in the lipid homeostasis and metabolism seem to be a hallmark of MS. To investigate the lipid profile can contribute to the better understanding of the pathophysiology of the disease (Pousinis et al., 2020). Moreover, this interesting class of compounds can be considered candidates for biomarkers of different aspects of MS, such as active and progressive phases of MS or potential target for new therapeutic approaches (Harlow et al., 2015). A summary of lipidomics studies performed on MS patients are reported in Table 5.

**Table 5:** Summary of lipidomics studies performed on MS patients

LIPIDOMICS CHARACTERIZATION OF MS SUBJECTS				
Sample	Year	Study	Platform	Metabolomic characterization
CSF	2014	Vidaurre et al.	LC-MS/MS	MS and Non-MS
	2014	Nogueras et al.	LC-MS/MS; GC-MS	MS and Non-MS
	2014	Pieragostino et al.	LC-MS/MS	MS and Other Neurological Diseases
SERUM	2011	Boccio et al.	LC-MS	MS, HC and Other Neurological Diseases
PLASMA	2009	Wilkins et al.	LC-MS/MS	MS
	2018	Kurts et al.	LC-MS/MS	MS and HC
	2021	Ferreira et al.	LC-MS	MS and HC
Brain tissue (Autopsy material)	2012	van Doorn et al.	LC-MS/MS	MS and HC

Previously, Del Boccio et al. through MS combined with LC, found a noticeably alteration in the lyso-phosphatidylcholine (LPC) and lyso-phosphatidylethanolamine (LPE) species: more in detail, MS patients showed reduced levels of LPC (16:0), LPC(18:0) and LPC(18:1), and increased concentrations of LPE (24:1). A decrease in LPC/PC ratio was also evidenced, indicating a specific trend in reduced LPC in MS (Del Boccio et al., 2011).

Later, an interesting study compared firstly the plasma lipidomic profile of control subjects and MS patients, and then the profile of patients affected by RRMS considering both the relapsing and the remitting states. Few PC species discriminated HC from MS: in particular, plasmalogens PC(P-38:6); PC(P-36:2)/PC(O-36:3); PC(P-36:4)/PC(O-36:5); PC(P-34:2)/PC(O-34:3); PC(P-36:5) and the diacyl species PC(34:4); PC(36:6); PC(32:2); PC(36:3); PC(36:5); PC(34:3); PC(34:2) and PC(38:1) were found decreased in the MS class. Only PC (38:1) was statistically different between the relapsing RRSM (where it was found increased) and the remitting RRMS (Ferreira et al., 2021).

Another study by Kurz J.et al. investigated the plasma sphingolipids profile of patients affected by SM founding significantly increased levels of different ceramides such as C16:0-Cer; C16:0-glucosylceramide (GlcCer); C18:0-LacCer; C18:0-GlcCer; C24:0-Cer; C24:1-Cer and C24:1-GlcCer and decreased level of C16:0-lactosylceramide (LacCer) (Kurz et al., 2018). Partially in line with these results, a study performed on CSF samples of MS patients through targeted LC-MS/MS approach revealed significantly increased levels of C16:0-Cer; C16:0-monohexosylCer and C24:0-Cer compared to control subjects.

Untargeted lipidomic analysis on CSF of patients with MS showed marked reductions of several phosphocholines (PC and lysoPC, LPC) and sphingomyelins (SM): PC(28:0), PC(28:1), PC(35:4), PC(36:1), PC(36:8), PC(37:6), LPC(18:1), LPC(20:4), SM [d18:0/16:1 (9Z) (OH)], SM(d18:1/14:0), SM(d18:1/16:0), SM(d18:2/20:0), SM(d18:2/22:1) and SM [d18:1/24:1 (15Z)] compared to the controls. On the other hand, PC (32:2) and PC(36:3) showed significantly higher levels in the same pathological samples compared to the control class (Pieragostino et al., 2018). The involvement of the sphingomyelin pathways may confirm disorders about the decompaction and destabilization of myelin structure (Jana and Pahan, 2010). Another study investigated the CSF lipidomic profile with the non-targeted approach as well, founding a decrease level of PC(P27:1), PS(40:3) and several triglycerides (TG) such as TG (37:2), TG (44:5), TG (50:1), TG (52:3), TG (55:5), TG (56:6), TG (57:4), TG (57:7), TG (58:1), TG (58:3), TG (59:6), TG (60:10), TG (61:8), TG (61:10), TG (62:8) in MS patients. It was also evidenced significantly increased concentrations of some fatty acids FA 20:0 and PC(25:2), PC(42:6), PE (21:0), TG (56:4), TG (57:6), TG (59:2), TG (63:8), TG (64:10), and some diglycerides DG (18:3), DG (32:2), DG (36:6), DG (38:6), DG (39:2), DG (42:5) (Nogueras et al., 2019).

The importance of the sphingomyelins in the MS disease was also proved by van Doorn et al. who investigated the effect of the sphingosine-1 receptor agonist, fingolimod, on sphingomyelin metabolism in active MS lesions. In the pathological context, astrocytes isolated from MS lesions expressed greater mRNA levels of the enzyme responsible for the ceramides production (ceramide-producing enzyme acid sphingomyelinase (ASM)) compared to astrocytes from control white matter. Interestingly, after the incubation of astrocytes with fingolimod, a reduction in ceramide production and mRNA expression of ASM was found (van Doorn et al., 2012).

The strength of the lipidomic analysis performed on CSF samples is that this biofluid is in direct contact with the CNS and can precisely reflects its changes related to pathological disfunctions (Vidaurre et al., 2014). CSF represents a great source of information but, on the other hand, it is often not possible to take it from healthy subjects for ethical reasons. Thus, limitations of the studies involved CSF are often related to the need for a direct comparison between MS and controls.

The studies previously investigated suggest specific alterations in MS lipid homeostasis and metabolism, which can be assessed through the serum/plasma/CSF lipidomic analysis. Despite the promising results obtained by the reviewed studies, some evidence did not find substantial differences between the plasma lipid profile of MS patients and controls (Wilkins et al., 2009).

More in general, lipidomic represents a valid tool to explore pathophysiological aspects still unclear in the MS scenario but considering the complexity of the “lipid sea” the biological interpretation of the results represents the best challenge. In this light, further studies are desirable to optimize the experimental process and to characterize MS lipid profile and metabolism.

## Multi-omics data integration

Access to large-scale omics datasets (genomics, transcriptomics, proteomics, metabolomics, and others) has revolutionized biology and led to the development of systems approaches to improvement our understanding of biological processes, including in MS (Misra et al., 2018). In this context, the integration of omics data is one of the main challenges in the era of precision medicine, in particular in complex disease like MS. Analyzing strengths and limitations of different omics approaches, if on one hand the interpretation of genome and transcriptome data in the context of biological function and phenotype is problematic (Lappalainen et al., 2013), on the other side combining data from proteomics and metabolomics with genomics and transcriptomics facilitate to overcome this limitation by providing molecular information that links genetic and epigenetic variation with phenotypic presentations. Beyond this, the limitations due to proteome and metabolome quantification, variability in sample handling, platform used, interindividual heterogeneity of different molecules quantification deserve to be mentioned (Spicer et al., 2017). Supplemental materials details aim, samples and analytical techniques of proteomics, metabolomics and lipidomics. The main challenge for integrating different omics datasets is to discern the true biological signal in the large number of observations per sample. Indeed, a genome typically includes millions of variants, a transcriptome a few thousand molecules, a transcriptome about 2000 molecules, and proteomes and metabolomes include thousands of quantified molecules (Misra et al., 2018)). This makes the omics experiment a computationally complex process, in which extracting meaningful correlations and true interactions is a difficult goal. Omics datasets closest to the genotype (genomics and transcriptomics) and those closest to the phenotype (proteomics and metabolomics) are integrated using statistical or advanced machine learning approaches, for a multi-omics view (Libbrecht & Noble 2015). In this way, machine-learning approaches, which represents a subfield of artificial intelligence and includes various algorithms to create an accurate model for predicting sample outputs, help clustering, association with disease measures and prediction of disease evolution (Li et al., 2016). To date, only a few studies have investigated the combined-omics approaches and the methods of integrating omics data still need to be further improved, to better distinguish predictive biomarkers. Previously, some studies have used comprehensive omics measurements, followed by integrated omics analysis to describe molecular variation in specific cancer types (Jiang et al., 2016; Kamoun et al., 2016). Later, by using both label-free and targeted proteomics, lipidomics, and metabolomics followed by data integration in human serum samples, a model on the reprogramming of organ functions induced by metastatic melanoma has been proposed (Muqaku et al., 2017), revealing novel insights into the basic biology of this disease. Looking to autoimmune diseases, a longitudinal study of the drug response at multi-omics levels was recently performed using the peripheral blood of patients with rheumatoid arthritis, and shown how an integrative analysis combining data from different molecular classes and detailed clinical parameters can lead to a better understanding of the molecular and cellular systems associated with drug treatment and disease severity (Tasaki et al., 2018). Similarly, the multi-omics approach can lead to the understanding of drug response in MS, as well as the mechanisms underlying its pathogenesis and evolution. Therefore, the future challenge is to integrate the different omics approaches into more composite models, despite several issues and limitations of integration data, in order to discern biomarkers of significance for MS from random noise of biological system (Misra et al., 2018).

## Conclusion

According to the modern vision of precision medicine, the challenge of the future will be to use integrative analytical approaches that combine clinical characteristics, MRI variables, and information from different omics approaches (genomics, epigenomics, transcriptomics, proteomics, and metabolomics) to improve the management of MS (Zagon and McLaughlin, 2017). Notoriously, the heterogeneity and biological complexity of MS may make the identification of a single biomarker difficult; thus, the combination of different types of data obtained from the omics approaches, using advanced computational methods and predictive models, can help maximize the predictive power of these biomarkers. Omics approaches can serve to distinguish among the subtypes of MS and identify when the disease changes and becomes progressive, with the objectives of better identifying the patients that can be administered the various immunotherapies, predicting the treatment efficacy and safety, and optimizing the allocation of resources and the therapeutic path.
