# Erratum: Omics approaches to understanding the efficacy and safety of disease-modifying treatments in multiple sclerosis

**DOI:** 10.3389/fgene.2023.1169919

**Published:** 2023-03-03

**Authors:** Frontiers Production Office

**Affiliations:** Frontiers Media SA, Lausanne, Switzerland

**Keywords:** multiple sclerosis, genomics, transcriptomics, proteomics, metabolomics, disease-modifying treatments, efficacy, safety

Due to a production error, each author’s first name and surname were erroneously interchanged. The corrected author list appears below.

“Lorena Lorefice, Maristella Pitzalis, Federica Murgia, Giuseppe Fenu, Luigi Atzori, Eleonora Cocco“

The original version of this article has been updated.

