# Supplementary material for: Omics approaches to understanding the efficacy and safety of disease-modifying treatments in multiple sclerosis
Source: Front Genet. 2023 Jan 30;14:1076421. doi: 10.3389/fgene.2023.1076421 (PMC9922720; doi:10.3389/fgene.2023.1076421)
Supplement: Supplementary file 1 [file Table1.doc]

**Supplemental material. Summary of aims, samples and** analytical techniques of proteomics, metabolomics and lipidomics

|  | **Proteomics** | **Metabolomics** | **Lipidomics** |
| --- | --- | --- | --- |
| **Aims** | to define the proteins/peptides profile of a biofluid/tissue | to define metabolites profile of a biofluid/tissue | to define lipidic profile of a biofluid/tissue |
| **Samples** | CSF, blood, saliva, tears, urine, other biofluids/tissues | CSF, blood, saliva, tears, urine, other biofluids/tissues | CSF, blood |
| **Analytical**  **Techniques** | chromatography-based techniques | gas chromatography coupled to mass spectrometry (GC–MS) | gas chromatography coupled to mass spectrometry (GC–MS) |
|  | enzyme-linked immunosorbent assay (ELISA) | liquid chromatography coupled with single-stage mass spectrometry (LC–MS) | liquid chromatography coupled with single-stage mass spectrometry (LC–MS) |
|  | gel electrophoresis | nuclear magnetic resonance (NMR) spectroscopy |  |
